# Open Repair of a 12-cm Posttraumatic Aneurysm of Right Subclavian Artery

**DOI:** 10.3389/fsurg.2017.00071

**Published:** 2017-11-24

**Authors:** Konstantinos Tigkiropoulos, Omiros Chalvatzoulis, Eleftherios Chalvatzoulis, Kyriakos Stavridis, Dimitrios Karamanos, Ioannis Lazaridis, Andreas Efstathiou, Nikolaos Saratzis

**Affiliations:** ^1^Vascular Unit, 1^st^ University Surgical Department, Aristotle University, Papageorgiou Hospital, Thessaloniki, Greece; ^2^Cardiothoracic Unit, Interbalkan Center, Thessaloniki, Greece

**Keywords:** subclavian artery aneurysm, posttraumatic aneurysm, open repair, innominate-axillary bypass

## Abstract

**Purpose:**

To present a rare case of a patient with a 12-cm posttraumatic right subclavian artery aneurysm successfully treated with aneurysmectomy and innominate-axillary bypass.

**Case report:**

A 54-year-old man presented to the emergency department due to progressive dyspnea and hoarseness of voice. His medical record was unremarkable except that he had right-sided pneumothorax and multiple rib fractures from a car accident 16 years ago. A chest X-ray showed a mass in the upper lobe of the right lung, and the patient was hospitalized for further investigation. A computed tomography (CT) with intravenous contrast of the thorax was performed, which depicted a giant aneurysm of the right subclavian artery. Vascular and cardiothoracic surgeons were consulted immediately, and the operation was scheduled. Aneurysmectomy and innominate-axillary bypass were performed. The patient had an uncomplicated progress and was discharged on 5 days followed by a single antiplatelet therapy and symptom-free.

**Conclusion:**

Posttraumatic subclavian artery aneurysm is a rare entity. Imaging of the thorax is essential for the diagnosis and surgical preparation of the patient. Open repair remains the gold standard therapy for subclavian artery aneurysm despite the improvements in endovascular surgery in such huge aneurysms.

## Introduction

Subclavian artery aneurysms are rare, and they account for 1% of all peripheral aneurysms (1). Based on their anatomic location, they are classified as intrathoracic and extrathoracic (2). Here, we report our experience with the repair of a 12-cm posttraumatic intrathoracic right subclavian artery aneurysm that has been successfully treated with aneurysmectomy and innominate-axillary bypass.

## Case Report

**Figure 1:**
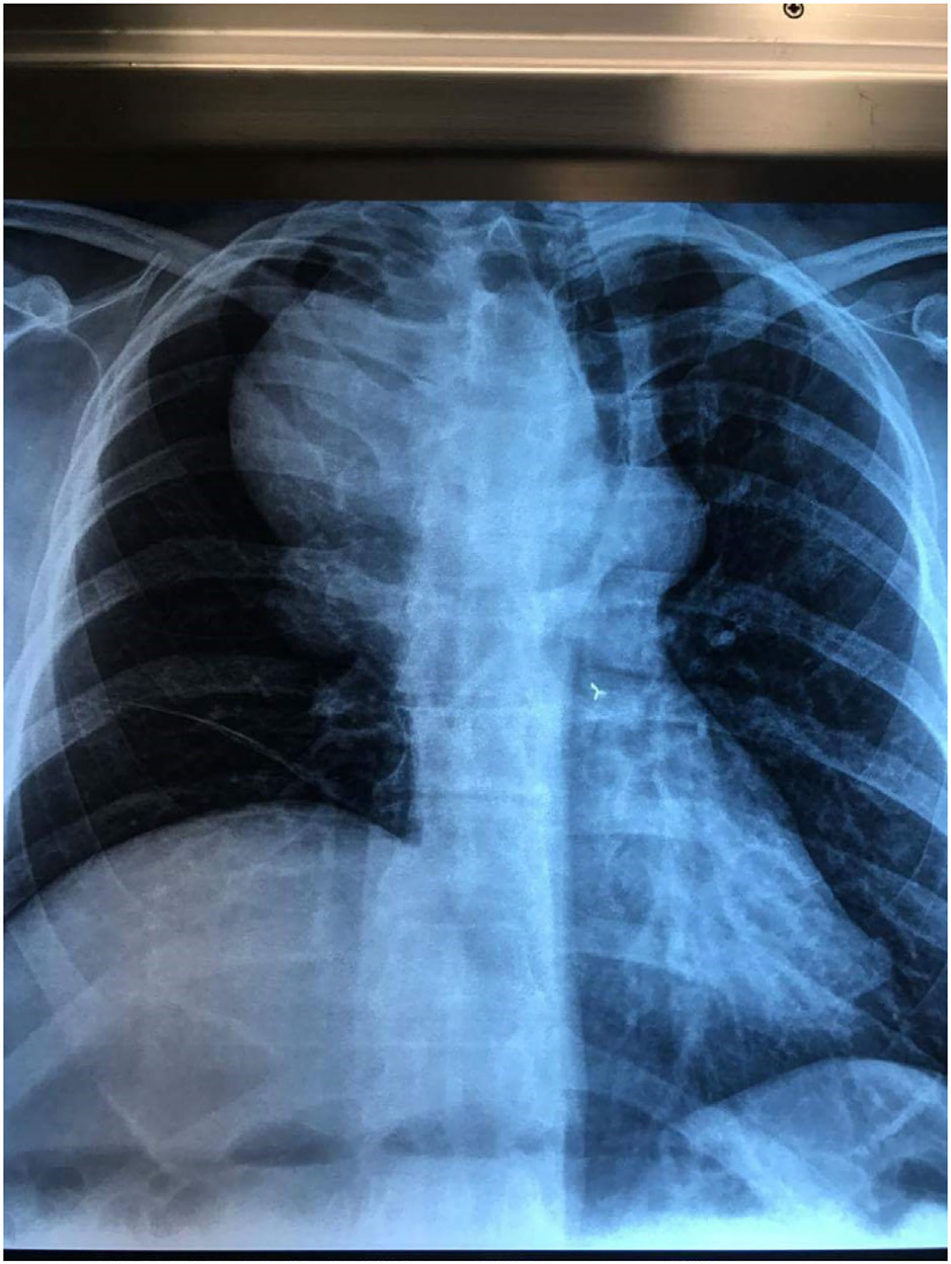
Chest X-ray shows a mass in the upper lobe of right lung with displacement of the trachea to the left and elevation of right diaphragm.

**Figure 2:**
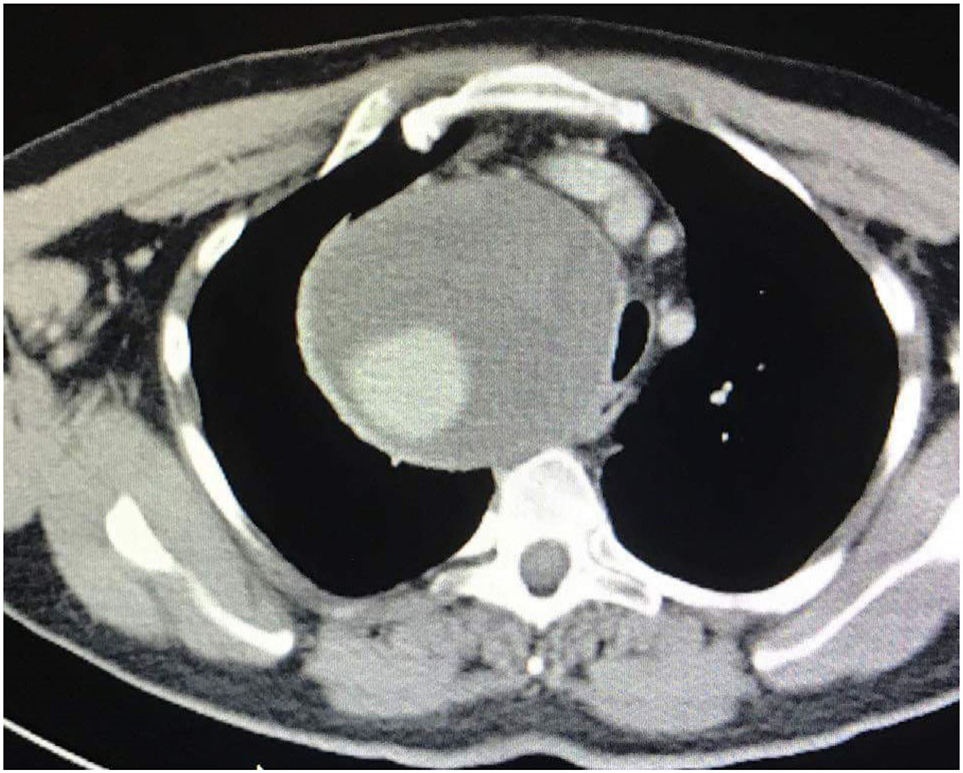
Axial-coronal computed tomography imaging shows a giant right subclavian aneurysm of 12 cm diameter.

**Figure 3:**
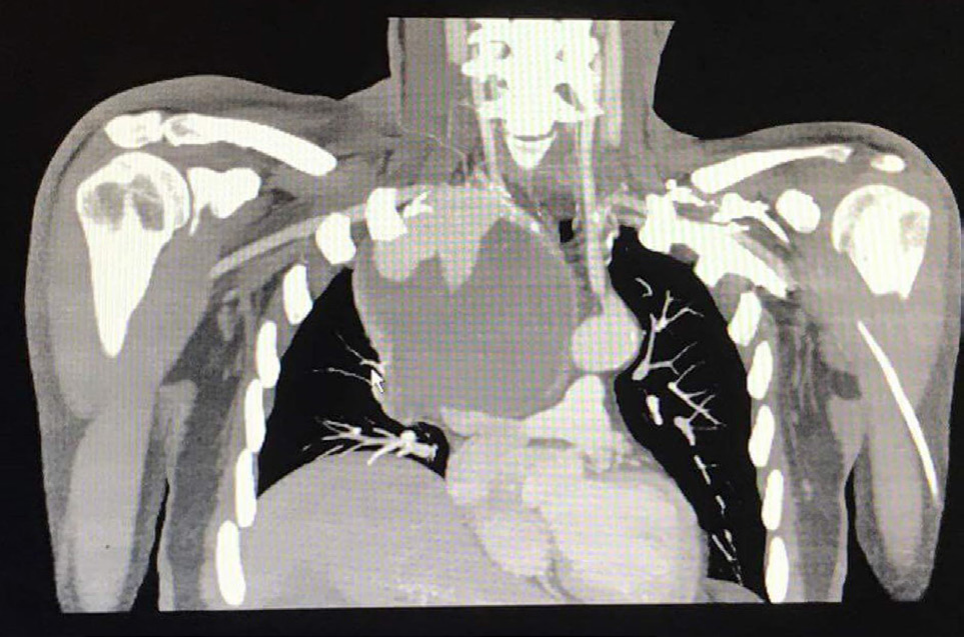
Axial-coronal computed tomography imaging shows a giant right subclavian aneurysm of 12 cm diameter.

A 54-year-old man presented to the emergency department complaining of dyspnea and gradually worsening hoarseness of voice. His medical history was free of any diseases except that he has been hospitalized for right-sided pneumothorax and multiple rib fractures from a car accident 16 years ago. Medical management included a tube thoracostomy in conjunction with analgesic medications. On admission, the results of the clinical examination and laboratory tests were normal. A chest X-ray was performed, which showed an intrathoracic mass in the upper lobe of the right lung with elevation of the right hemidiaphragm and displacement of trachea to the left (Figure 1). A provisional diagnosis of lung tumor was made, and the patient was hospitalized for further investigation. A computed tomography (CT) with intravenous contrast of the thorax was performed, which showed a giant aneurysm, 12 cm diameter, of right subclavian artery that displaced esophagus, trachea, aortic arch, and supra-aortic trunks to the left mediastinum (Figures 2 and 3). Vascular and cardiothoracic surgeons were consulted immediately, and the operation was scheduled.

## Surgical Management

Under general anesthesia, a right infraclavicular incision was first performed. After dissection of the pectoralis major muscle, opening of the clavipectoral fascia, and partial division of the pectoralis minor muscle, access to the right axillary artery was gained to obtain distal control of the aneurysm. Subsequently, a median sternotomy with extension of the incision to the right cervical area was done. The pericardial sac was opened, and the mediastinal adipose tissue was divided. The right sternocleidomastoid muscle was sharply dissected, and the right common carotid artery was recognized. An excellent exposure to the left/right innominate veins, superior vena cava, ascending aorta, innominate bifurcation, and right common carotid artery was achieved. The anteromedial wall of the aneurysm was detected at the right upper mediastinum. The proximal end of the aneurysm was l cm away from the origin of the subclavian artery. It was closely adherent to the proximal end of the right common carotid artery.

**Figure 4:**
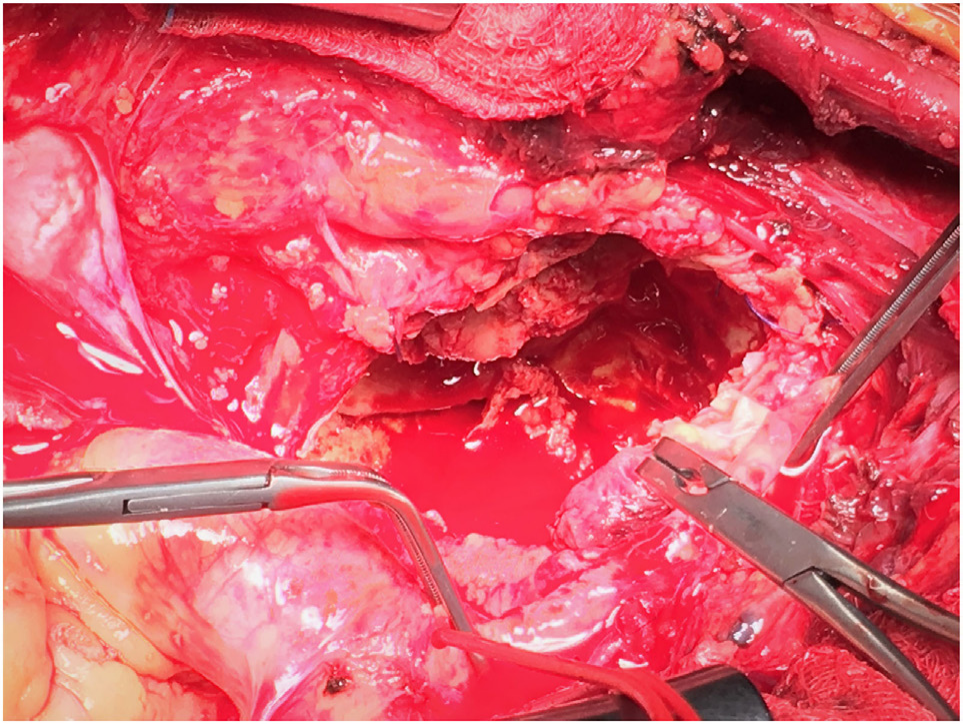
Aneurysmatectomy with removal of thrombus and backbleeding from subclavian branches.

**Figure 5:**
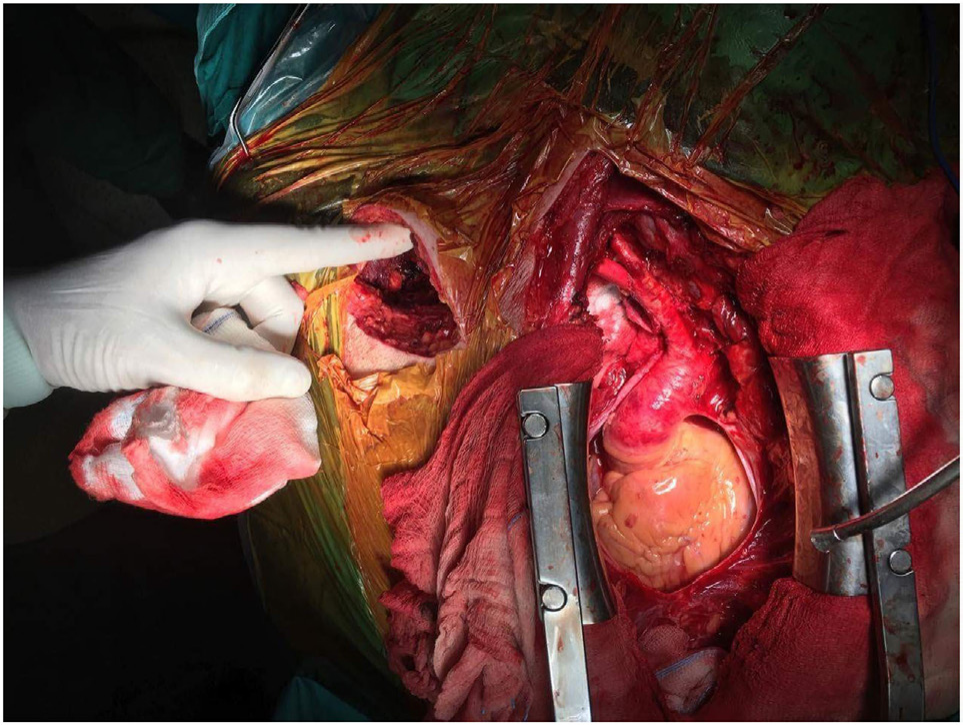
Median sternotomy with right infraclavicular incision. Note the PTFE graft at the origin of right subclavian artery.

After systemic heparinization and blocking of innominate, right common carotid, and axillary arteries, aneurysm was incised. Thrombus was removed from the sac, and hemorrhage from the right vertebral artery and costocervical trunk was revealed. Suture of the ostial branches was performed (Figure 4). After control of bleeding, an end-to-end anastomosis with 8 mm PTFE graft between the ostium of right subclavian artery and axillary was successfully performed (Figure 5). Patency was achieved with good flow. A thoracostomy tube was placed in the mediastinum with the tip near the aneurysmal sac, and the patient was transferred to the intensive care unit. He was hemodynamically stable with good distal perfusion of right upper extremity. Next day, radial systolic blood pressures were similar at both upper limbs. The patient was discharged after 5 days under single antiplatelet therapy. At follow-up 1 month later, the patient’s symptoms have been improved with no difficulty in breathing, good motivation of right upper extremity, and a chest X-ray depicted shrinkage of the aneurysm.

## Discussion

Subclavian artery aneurysm is a rare entity accounting for 1% of all peripheral aneurysms (1). Depending on the location of the aneurysm, they are classified as intrathoracic and extrathoracic (2). Atherosclerosis is the main cause of intrathoracic aneurysms followed by infection, injuries, cystic medial degeneration, Takayasu arteritis, and Marfan’s syndrome whereas thoracic outlet syndrome (TOS) or iatrogenic injuries are responsible for extrathoracic aneurysms (3, 4). Clinical presentation varies from asymptomatic mass to compression of the neighboring anatomic structures or distal embolization. Dysphagia, dyspnea, hemoptysis, and hoarseness of voice from esophageal, tracheal, lung, phrenic, and right recurrent laryngeal nerve compression have been described (5). Thoracic CTA is the preferred imaging modality since it helps for diagnosis and operational approach to subclavian artery aneurysm.

Vierhout et al. (5) in a 2010 review found 394 cases of subclavian artery aneurysm in 381 patients. Half of them (51%) presented with pulsatile mass, shoulder pain, and non-specific chest pain. Major complications such as embolization, rupture, and thrombosis were presented in 16%, 9% and 6% of the patients, respectively. Open and endovascular therapies had complication rates 28% and 26%, respectively, whereas mortality was similar in both types of repair (5%).

Our patient presented with dyspnea and hoarseness of voice due to right phrenic nerve paresis, tracheal and right laryngeal nerve compression. Constrast-enhanced computed tomography of thorax was the imaging modality of choice. Elective surgical repair was the treatment of choice since there was an increased risk of complications such as thrombosis, rupture, and embolization (4, 6). Endovascular repair was not feasible because proximal landing zone of the aneurysm was inadequate for proper deployment of the stent graft. Surgical approach included medial sternotomy with extension to right cervical area and infraclavicular incision. The origins of the great vessels were carefully dissected and controlled to obtain a proximal control of the sac whereas distal control was achieved by axillary artery dissection and blockage. After incision of the sac, suturing and ligation of the vertebral artery and other branches were necessary to control hemorrhage and to safely perform the bypass with the synthetic graft. Reimplantation of the vertebral artery was not feasible due to anatomic distortion of the right upper mediastinum by the aneurysm.

It must be noted that successful surgical repair was a collective process depending on the general anesthesia, surgical repair, intra-postoperative hemodynamic stability, and intensive care therapy.

## Conclusion

Posttraumatic subclavian artery aneurysm is a rare entity. To the best of our knowledge, this is first study to report the largest aneurysm of subclavian artery that had been repaired. Thoracic CTA imaging is essential for diagnosis and surgical preparation of the patient. Open repair remains the gold standard therapy for subclavian artery aneurysm despite the improvements in endovascular therapy.

## Consent

The written informed consent has been obtained from the patient for the publication of this case report. This case report was conducted in accordance with ISO14155:2011, Clinical investigation of Medical Devices for human Subjects-Good Clinical Practice, and the ethical principles that have their origin in the Declaration of Helsinki.

## Author Contributions

KS (literature review), DK (literature review), IO (literature review), OC (heart surgeon at operation), EC (heart surgeon at operation), KT (vascular surgeon at operation), and NS (vascular surgeon at operation).

## Conflict of Interest Statement

The authors declare that the research was conducted in the absence of any commercial or financial relationships that could be construed as a potential conflict of interest.
